# Comparison Study of Voltammetric Behavior of Muscle Relaxant Dantrolene Sodium on Silver Solid Amalgam and Bismuth Film Electrodes

**DOI:** 10.1155/2017/3627428

**Published:** 2017-09-13

**Authors:** Renáta Šelešovská, Pavlína Martinková, Michaela Štěpánková, Tomáš Navrátil, Jaromíra Chýlková

**Affiliations:** ^1^Faculty of Chemical Technology, Institute of Environmental and Chemical Engineering, University of Pardubice, Studentská 573, 53210 Pardubice, Czech Republic; ^2^J. Heyrovsky Institute of Physical Chemistry of the CAS, v.v.i., Dolejškova 3, 182 23 Prague 8, Czech Republic

## Abstract

Voltammetric behavior of muscle relaxant dantrolene sodium (DAN) was studied and the voltammetric methods for its determination using polished and mercury meniscus modified silver solid amalgam electrodes (p-AgSAE and m-AgSAE) as well as using bismuth film electrode (BiFE, ex situ plating on GCE) have been proposed. These working electrodes represent the most commonly used alternatives to mercury ones which come wrongfully into disfavor because of alleged toxicity of mercury. Within this work, the obtained results of DAN determination have been completed by corresponding statistical parameters and also some electrochemical characteristics of AgSAEs and BiFE were assessed, especially in comparison with the mercury electrodes.

## 1. Introduction

In the area of electroanalytical chemistry, mercury electrodes present a tool with unique properties, especially with high hydrogen overvoltage and very well and easy renewable surface. However, the restrictions [1] connected with the use of liquid mercury are often discussed. Therefore, the development of new electrode materials belongs to long-term trends in electroanalytical chemistry. Amalgam as well as bismuth electrodes are among the most often mentioned alternatives to the mercury electrodes. It is especially due to their width potential windows in cathodic area which allow measuring of reduction reactions.

Solid amalgam electrode was introduced in 2000 [2, 3]. It combines the advantages of mercury and solid electrodes, for example, the high hydrogen overvoltage, good mechanical stability, and utilization of nontoxic material [4]. Beside the classical type, they are nowadays used in various forms, like, for example, paste [5], composite [6], or crystalline [7]. Silver solid amalgam electrode (AgSAE) is the most commonly used type of amalgam electrode. Surface of AgSAE could be polished (p-) [8] or modified by mercury meniscus (m-) or thin film (MF-) [4]. Particularly the types of p-AgSAE and m-AgSAE have been widely used in practice in inorganic [8–11] and organic analysis [12–18] as well as in analysis of DNA and peptides [19–22]. The universality is the biggest advantage of AgSAEs. Bismuth electrodes were described also in 2000 [23] as the electrodes based on electrolytically deposited film of Bi, on a disc of metal Bi, or on another electrode material with the blended Bi powder. Bi film electrodes (BiFE) are the most often used form [24]. Glassy carbon electrodes (GCE) [23, 24], various metal electrodes (ME) [25], screen printed electrodes (SPE) [26, 27], and AgSAE [28, 29] are frequently used as substrates and the Bi film can be deposited in mode ex situ or in situ [30–32]. Most publications devoted to the application of BiFE are focused on determination of metal cations [27, 30, 33–37] and the number of papers dealing with analysis of organic compounds is relatively low [38–40].

Dantrolene sodium (DAN) was chosen as an analyte for the following study focused on comparison of electrochemical properties of p-AgSAE, m-AgSAE, and BiFE. DAN (1-{[5-(4-nitrophenyl)-2-furyl]methylideneamino}imidazolidine-2,4-dione, CAS: 7261-97-4) was firstly described in 1967 as one of hydantoin derivatives proposed as a muscle relaxant [41]. It is the drug used for treatment and prevention of malignant hyperthermia, muscle spasticity, or neuroleptic malignant syndrome [42]. The most widely used method for DAN determination is high-performance liquid chromatography with UV-Vis detectors [43–46].

DAN voltammetric behavior on mercury electrodes was described in [47–49]. Cox et al. [47] applied DPP with dropping mercury electrode (LOD 3.0 × 10^−7^ mol L^−1^). Similar method was presented by C. S. Reddy and S. J. Reddy [48] (LOD 8.0 × 10^−8^ mol L^−1^). Other authors applied various polarographic and voltammetric techniques and the lowest LOD obtained using adsorptive stripping square wave voltammetry (AdS SWV) (2.1 × 10^−10^ mol L^−1^) [49]. These papers describe the reduction of DAN via the reduction of the present nitro group (−0.25 V and −0.50 V versus SCE) and reduction of the azomethine group (−0.85 V) in acidic medium. In alkaline medium, only the first cathodic signal corresponding to the reduction of –NO_2_ group can be observed [47–49]. DPP was also applied for study of physicochemical properties of DAN, especially for the determination of its p*K*_a_ value [50]. Authors Hendawy et al. used SWV in connection with GCE (LOD 1.1 × 10^−7^ mol L^−1^) and with pencil graphite electrode (LOD 3.5 × 10^−7^ mol L^−1^) [51]. Our working group published results dealing with the application of boron-doped diamond electrode (BDDE) for DAN determination (LOD 1.0 × 10^−7^ mol L^−1^) [52].

The subject of this work is to examine voltammetric behavior of DAN on p-AgSAE, m-AgSAE, and BiFE (ex situ plating on GCE) and to develop sensitive methods for its precise determination. The main goal is to compare the electrochemical properties of amalgam and bismuth electrodes as the commonly used alternatives to mercury electrodes. Some preliminary results related to this study were published as a poster on electroanalytical conference ESEAC 2016 [53].

## 2. Experimental

### 2.1. Reagents and Materials

All chemicals used for preparation of standard solutions, supporting electrolytes and other stock solutions, were of p.a. purity. The standard solution of DAN (98%, Sigma-Aldrich, Czech Republic) was prepared by dissolution in methanol (Penta-Švec, Czech Republic). Britton-Robinson buffer (BRB) was prepared by mixing of alkaline component (0.20 mol L^−1^ NaOH (Lachema, Brno, Czech Republic)) and acidic component (0.04 mol L^−1^ H_3_PO_4_, H_3_BO_3_, and CH_3_COOH (all Lachema, Brno, Czech Republic)). The electrolytes of H_2_SO_4_ were prepared from 96% H_2_SO_4_ (Penta-Švec, Czech Republic). KCl powder (Lachema, Brno, Czech Republic) was dissolved in the distilled water. Acetate buffer (AB, pH 4.5) was created from sodium acetate and acetic acid (both from Lachema, Brno, Czech Republic). The stock solution of Bi^3+^ (10 mg L^−1^, Analytica Co. Ltd., Prague, Czech Republic) served for Bi film deposition.

### 2.2. Instrumentation

All measurements were performed by computer controlled Eco-Tribo Polarograph [54] (Polaro-Sensors, Czech Republic) with the software POLAR.PRO in a three-electrode setup, where HMDE (surface area 0.73 mm^2^), m-AgSAE (0.39 mm^2^), and p-AgSAE (0.28 mm^2^) originating from EcoTrend Plus, Czech Republic, and BiFE (7.07 mm^2^) deposited on GCE substrate (Monokrystaly, Czech Republic) served as the working electrodes. Saturated silver/silver chloride electrode was used as the reference and platinum wire as the auxiliary electrode (Monokrystaly, Czech Republic). The experiments were realized at laboratory temperature (23.0 ± 2.0°C). Oxygen was removed from the solutions by N_2_ (purity class 4.0; Linde, Czech Republic) bubbling for five minutes before analysis. The nitrogen atmosphere was maintained above the analyzed solution during the whole analysis. The pH measurements were performed using pH-meter Accumet AB150 (Fisher Scientific, USA) and solutions of DAN were prepared in an ultrasonic bath Bandelin Sonorex (Schalltec, Germany). The linear least-square regression in OriginPro 9 (OriginLab Corporation, USA) was used for the evaluation of calibration curve and the relevant results (slope and intercept) were reported with confidence interval for 95% probability. The limits of detection and quantification (LOD and LOQ) were calculated as three and ten times the standard deviation for the blank solution divided by the slope of the calibration curve.

### 2.3. Procedures

#### 2.3.1. Voltammetric Measurements

Cyclic voltammetry (CV) and direct current voltammetry (DCV) were used for the examination of voltammetric behavior of DAN in dependence on pH and on the scan rate (*v*). Differential pulse voltammetry (DPV) was applied for DAN determination using HMDE, m-AgSAE, p-AgSAE, and BiFE as well. The parameters of this method were optimized for each used electrode individually and are summarized in Table 1. DPV peaks were evaluated from the straight line connecting the minima before and after the peak.

#### 2.3.2. Preparation of AgSAE

Before the first application, the surface of p-AgSAE was abraded on a soft emery paper followed by polishing on the polishing kit (Electrochemical Detectors, Czech Republic), which consisted of the polyurethane pad, alumina suspension (particle size 1.1 *μ*m), and alumina powder (0.50 *μ*m). The polishing was made once or twice a week in case of a long-term measurement. After polishing, on the beginning of every day or after pause longer than one hour, the activation of the p-AgSAE was carried out in the stirring solution of 0.20 mol L^−1^ KCl at potential −2200 mV for 5 min. By this way, the p-AgSAE was ready for measurements. The m-AgSAE is prepared from p-AgSAE by immersion of the electrode top into liquid mercury. The created mercury meniscus should be renewed usually once a week. The surface of both modifications of AgSAE was electrochemically regenerated between measurements directly in analyzed solutions by insertion of negative potential value of −1200 mV for 20 s.

#### 2.3.3. Preparation of BiFE

GCE was utilized as a substrate for Bi film deposition. It was firstly polished using above-mentioned polishing kit, rinsed with methanol, left in ultrasonic bath for 3 min., and rinsed by distilled water. Bi film was deposited on surface electrolytically in mode ex situ, that is, from the 10 mL of 0.10 mol L^−1^ solution of AB (pH 4.5) containing 100 *μ*L Bi^3+^ solution (10 mg L^−1^). The plating conditions were first taken from literature: potential of deposition (*E*_dep_) −1000 mV, time of deposition (*t*_dep_) 60 s [55]. During the experiments these parameters were optimized for DAN determination: *E*_dep_  −1000 mV; *t*_dep_ 100 s. The new Bi film was prepared on the GCE surface after 30 scans due to the narrowing the potential window, worsening of repeatability, and decreasing of the observed signals. The repeatability of measured peaks was slightly improved by insertion of the similar regeneration step as in case of AgSAEs (*E*_reg_ = −1200 mV; *t*_reg_ = 20 s).

#### 2.3.4. Analysis of Spiked Drinking Water

Model samples of drinking water spiked with DAN on two concentration levels were analyzed using each particular tested electrode. Drinking water was sampled from the tap in the laboratory of the University of Pardubice. Samples were spiked with DAN stock solution to the following concentrations: 1.0 × 10^−7^ and 1.0 × 10^−8^ mol L^−1^ (m-AgSAE; BiFE); 2.0 × 10^−6^ and 2.0 × 10^−7^ mol L^−1^ (p-AgSAE). The analyses proceeded in the cell containing 10 mL of sample with addition of 1.0 mL of BRB. The standard addition method with at least 2-3 additions was applied. Each determination was 5 times repeated and the average value with confidence interval and RSD was calculated.

## 3. Results and Discussion

**Figure 1:**
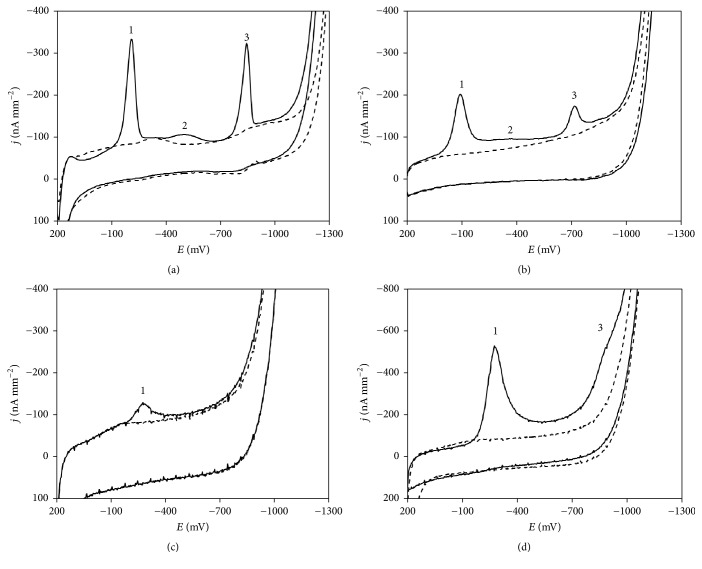
CV voltammograms of 5.0 × 10^−6^ mol L^−1^ DAN recorded on m-AgSAE (a), p-AgSAE (b), and BiFE (c). Electrolyte: BRB (pH 6.0); *E*_in_ = +200 mV; *E*_fin_ = −1300 mV; *v* = 100 mV s^−1^; CV voltammogram of 5.0 × 10^−5^ mol L^−1^ DAN recorded on BiFE (d); dashed lines: supporting electrolyte; solid lines: after the addition of DAN.

At the beginning, CV voltammograms in BRB of pH 6.0 were measured using HMDE, m-AgSAE, p-AgSAE, and BiFE before and after the addition of 5.0 × 10^−6^ mol L^−1^ DAN. The obtained curves for p-AgSAE, m-AgSAE, and BiFE are shown in Figure 1. Due to different surface areas of the electrodes, the obtained current results (*I*; *I*_*p*_/nA) were recalculated to the current densities (*j*; *j*_*p*_/nA mm^−2^). The cathodic limit of the potential window very similar to that specified for HMDE is one of the most often mentioned advantages of AgSAEs as well as of BiFE. For the comparison of this parameter, the cathodic potential limit was defined as potential value at which the *j*_*p*_ reaches the level of −500 nA mm^−2^. The widest cathodic potential window to the value of −1370 mV versus Ag/AgCl/KCl (sat.) was recorded using HMDE. Using the amalgam electrodes, the applicable cathodic potential in BRB (pH 6.0) was −1295 mV (m-AgSAE) and −1120 mV (p-AgSAE) (dashed lines in Figure 1). This implies that the hydrogen overvoltage gradually decreases from the HMDE via m-AgSAE, down to p-AgSAE; this phenomenon is probably caused by the decrease of mercury content. Both types of amalgam electrodes exhibit wider potential window than BiFE.

After the addition of DAN to the electrolyte of pH 6.0 (in accordance with [47–49]) three reduction signals have been registered using HMDE. Similar voltammetric curves with three cathodic peaks were obtained also with p-AgSAE and m-AgSAE (solid lines in Figures 1(a) and 1(b)). Using BiFE, only the first signal corresponding to the reduction of the nitro group was registered (Figure 1(c)). As it can be seen in the Figure 1(d), DAN concentration must increase ten times to record the peak 3 corresponding to the reduction of azomethine group. This signal is largely overlapped with hydrogen evolution signal. The second reduction signal of –NO_2_ was not observed on BiFE. The peak potentials (*E*_*p*_) of the particular DAN signals registered at all compared electrodes are summarized in Table 2. It is evident that the electrodes with liquid mercury surface, that is, HMDE and m-AgSAE, have provided very similar *E*_*p*_ values. It confirms realization of the same electrode processes as well as the similarity in electrochemical properties, for example, the charge transfer coefficients. Contrary to common experiences with amalgam electrodes, all DAN peaks obtained on p-AgSAE were significantly shifted to more positive potentials in comparison with HMDE and m-AgSAE suggesting easier realization (smaller energetic barrier; better charge transfer coefficient) of the electrode reactions on the solid amalgam surface. On the other hand, BiFE provided peaks at more negative potential values. This shift corresponds to worse charge transfer coefficient and to the necessity to overcome a higher energetic barrier on BiFE.

Figure 1 offers also a comparison of the current densities (*j*_*p*_) of the particular DAN signals registered using different electrodes. It is evident that the highest values of *j*_*p*_ were achieved using m-AgSAE. This is true even for comparison with mercury electrode when the value of *j*_*p*_ for peak 1 of DAN was evaluated as −127 nA mm^−2^ for HMDE and −235 nA mm^−2^ for m-AgSAE. Current densities achieved with p-AgSAE were lower and the values recorded with BiFE were the lowest. No oxidation signals were recorded on all tested electrodes suggesting the irreversible character of the observed electrode processes.

### 3.1. The Effect of Supporting Electrolyte pH

**Figure 2:**
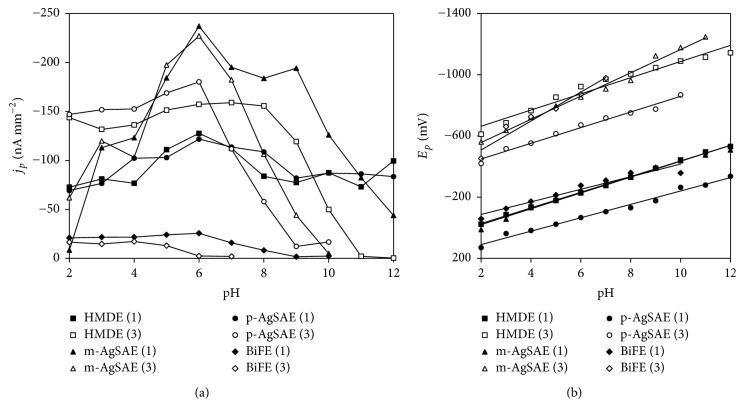
Dependence of *j*_*p*_ (a) and *E*_*p*_ (b) of DAN signals on pH of electrolyte obtained on all tested electrodes. Method: CV; electrolyte: BRB (pH 2.0–12); *E*_in_ = +200 mV; *E*_fin_ = −1300 mV; *v* = 100 mV s^−1^; *c*_DAN_ = 5.0 × 10^−6^ mol L^−1^; (1) peak 1; (3) peak 3.

The influence of pH of supporting electrolyte (BRB of pH 2.0–12) on the electrochemical behavior of 5.0 × 10^−6^ mol L^−1^ DAN was studied using CV. The obtained dependence of current densities of DAN signals on pH is depicted in Figure 2(a). Only peaks 1 and 3 were evaluated because they were recorded on all electrodes. It is seen from Figure 2(a) that the peak 1 was recordable in the whole tested pH range from 2.0 to 12 on HMDE and on both AgSAEs. Using BiFE, this signal was obtained only up to pH 10. Signal 3 was obtained usually in narrower pH range with exception of HMDE. It is evident that the highest values of *j*_*p*_ were achieved in slightly acidic and neutral media for both signals and all electrodes. Therefore, BRB of pH 6.0 (HMDE; m-AgSAE) and of pH 5.0 (BiFE), respectively, was selected as a supporting electrolyte for the following measurements.

It is obvious from Figure 2(b) that both DAN signals were shifted to negative potentials with increasing pH. This trend corresponds with the participation of protons in the electrode reactions. The plotted dependence of *E*_*p*_ on pH is linear and its parameters are summarized in Table 3. The slope values especially for the first signal are rather close to the theoretical value from Nernst equation (−0.059 V). Therefore, it is possible to conclude that the reaction mechanism involves the same number of the electrons and protons.

### 3.2. The Effect of Scan Rate and Elimination Voltammetry with Linear Scan

**Figure 3:**
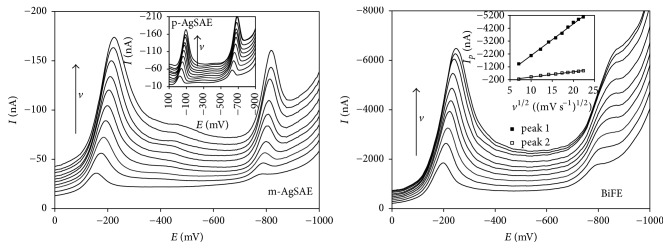
DC voltammograms of 5.0 × 10^−6^ mol L^−1^ DAN recorded on m-AgSAE, p-AgSAE, and BiFE in dependence on scan rate. Electrolyte: BRB (pH 6.0 (AgSAEs) and 5.0 (BiFE)); *E*_in_ = +200 mV, *E*_fin_ = −1200 mV; *v* = 50–500 mV s^−1^.

The influence of *v* on voltammetric behavior of DAN (5.0 × 10^−6^ mol L^−1^) was investigated in BRB of pH 6.0 for mercury based electrodes and in BRB of pH 5.0 for BiFE applying DCV. The obtained voltammograms for tested amalgam and bismuth electrodes are shown in Figure 3. It was found that both observed signals (1 and 3) recorded on HMDE and m-AgSAE increased linearly with increasing *v*, which corresponds to the adsorption-controlled processes. Nevertheless, as can be concluded from parameters of the “log(*I*_*p*_)_log(*v*)” dependence summarized in Table 4, the electrode processes are more complicated. The value 1.0 is not included in the confidence intervals of the slopes for HMDE (0.868 ± 0.023) and for m-AgSAE (0.835 ± 0.011). It is possible to conclude that the processes are controlled by the adsorption with weak participation of diffusion.

In the case of p-AgSAE and of BiFE, *I*_*p*_ of both DAN signals also increases with increasing *v*, but the increments are not directly proportional. A linear dependence between *I*_*p*_ and *v*^1/2^ has been obtained, how it is evident from Table 4. Thus, it can be assumed that these cathodic processes are diffusion-controlled. Nevertheless, the value of 0.5 is not included in the confidence intervals of slopes calculated for reduction peak 1 (p-AgSAE, BiFE) as well as for peak 3 (p-AgSAE). It indicates a very slight influence of adsorption on the ongoing reduction processes. The peak 3 recorded on BiFE seems to be completely diffusion-controlled with the value of 0.5 included in the confidence interval.

**Figure 4:**
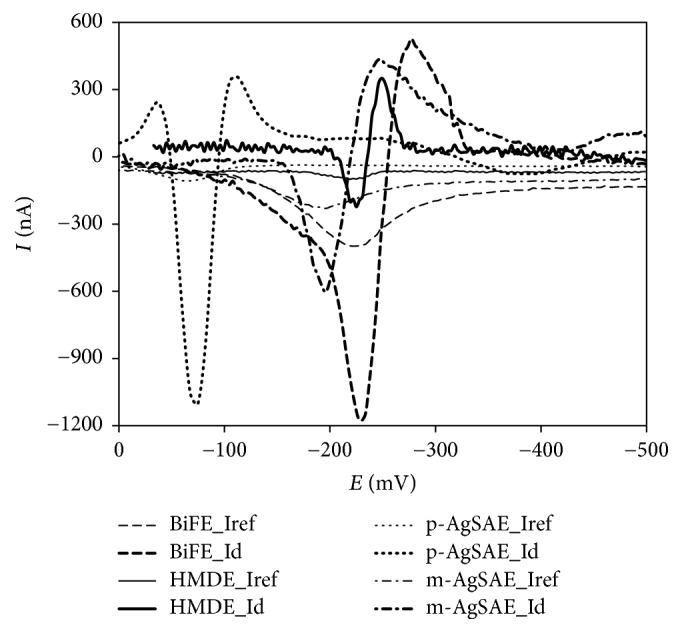
Elimination voltammograms of DAN recorded on tested electrodes. *c*_DAN_ = 5.0 × 10^−6^ mol L^−1^. DC curves registered under optimized conditions. Elimination functions calculated from scan rate set: 50, 100, 200, and 400 mV s^−1^. *I*_ref_: 200 mV s^−1^, *I*_*d*_: diffusion current after elimination of kinetic, capacity (adsorption), and irreversible components; these functions were calculated according to (31) from [56].

Using elimination voltammetry with linear scan (EVLS) [56–59], it was possible to reveal the reaction mechanisms in more detail. It was confirmed that all three peaks registered using HMDE correspond to the reduction processes realized in adsorbed state. However, the ratios of the elimination peak heights to elimination counterpeak heights did not correspond to the theoretical values (i.e., about 3 : 4 [60]) (Figure 4). Nevertheless, the values (about 0.6) confirmed strong influence of adsorption on the reduction process. It is in fair correspondence with previously mentioned results. Reduction process corresponding to the peak 1 on the m-AgSAE could be understood as reduction in a bit weakly adsorbed state (the ratios of the elimination peak heights to elimination counterpeak heights amounted to 1.1 : 1) (Figure 4). In the case of the peak 3, it can be concluded that the reaction process can be described as reduction process in very strongly adsorbed state. Both these conclusions are in good agreement with above described scan rate and log-log tests. In the case of p-AgSAE, the EVLS revealed that the reduction processes are more complicated. Due to the presence of the counterpeak situated before and the small counterpeak situated after the main elimination peak, it is possible to conclude that both reduction processes are preceded by kinetically controlled processes and both are realized in very weakly adsorbed state. Reduction process corresponding to the peak 1 on the BiFE could be understood as reduction in weakly adsorbed state (the ratios of the elimination peak heights to elimination counterpeak heights amounted to 2 : 1) (Figure 4). The reactions corresponding to the process represented by peak 3 cannot be completely revealed by EVLS due to overlapping with hydrogen evolution signal.

### 3.3. Analytical Application

#### 3.3.1. Electrode Surface Regeneration

DPV was applied for the DAN determination in model solutions. While the HMDE represents electrode with easily renewable surface, AgSAEs and BiFE require insertion of some pretreatments and regeneration steps. AgSAEs require a renovation of the surfaces (by polishing or via forming of mercury meniscus) once a week and electrochemical activation in 0.20 mol L^−1^ (*E* = −2200 mV; *t* = 300 s) after longer inactivity (a few hours). Two procedures of electrochemical regeneration between measurements were tested: (a) application of constant negative potential for 20 s; (b) insertion of thirty potential “jumps” between positive and negative potential values (limiting potentials were kept for 0.30 s). Application of −1200 mV for 20 s was found to be the most appropriate for the regeneration of p-AgSAE and m-AgSAE as well. Correctness of this choice was confirmed by a low values of the relative standard deviation of 11 repeated measurements (RSD_11_ < 3.0%) obtained for DAN concentration of 5.0 × 10^−7^ (m-AgSAE) and 2.0 × 10^−6^ (p-AgSAE) mol L^−1^.

**Figure 5:**
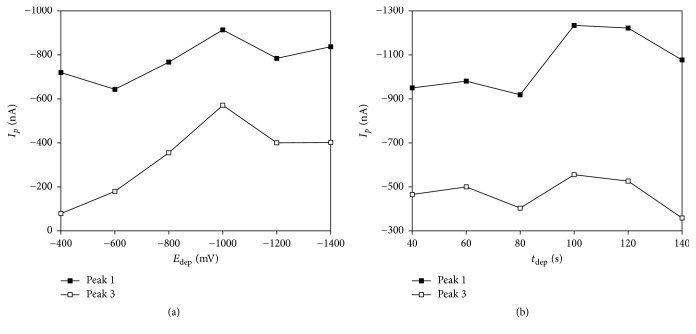
Optimization of *E*_dep_ (a) and *t*_dep_ (b) of Bi film deposition. Method: DPV; electrolyte: BRB (pH 5.0); *E*_in_ = +200 mV; *E*_fin_ = −1100 mV; *v* = 40 mV s^−1^;* pulse height* = −50 mV;* pulse width* = 60 ms; *c*_DAN_ = 2.0 × 10^−5^ mol L^−1^; plating solution: AB (pH 4); *E*_dep_ = −400–−1400 mV (a) and −1000 mV (b); *t*_dep_ = 120 s (a) and 40–140 s (b).

The thin Bi film on GCE was prepared ex situ in solution of AB (pH 4.5) containing 100 *μ*L of 10 mg L^−1^ Bi^3+^ solution. The plating conditions, namely, potential (*E*_dep_) and time (*t*_dep_) of deposition, were firstly taken from literature [55] and, subsequently, experimentally optimized specifically for DAN determination. The procedure of the optimization was as follows: previously used Bi film was removed from GCE by insertion of +400 mV for 15 s in an acidic solution. The substrate was rinsed as described in* Experimental part*. The ex situ electroplating of the Bi film was performed in an air-saturated plating solution under the changing conditions. Thus prepared BiFE was used for measurement of 2.0 × 10^−5^ mol L^−1^ DAN solution. Firstly, the dependence of *I*_*p*_ of DAN on *E*_dep_ in the range from −400 to −1400 mV was studied (*t*_dep_ = 180 s). As illustrated in Figure 5(a), the highest *I*_*p*_ was observed for *E*_dep_ close to −1000 mV. After that, the influence of *t*_dep_ was investigated in range from 40 to 140 s (*E*_dep_ = −1000 mV). In this case (Figure 5(b)), the optimal *t*_dep_ was found as 100 s. Without any regeneration process BiFE provided RSD_11_ 6.41% (2 × 10^−5^ mol L^−1^ DAN). The repeatability of measured signal was improved (RSD_11_ = 2.27%) by insertion of the similar regeneration step as in case of AgSAEs (*E*_reg_ = −1200 mV; *t*_reg_ = 10 s). The Bi film was removed and newly prepared on the GCE surface after every 30 records due to the narrowing the potential window, worsening of repeatability, and decreasing of the observed current signals.

#### 3.3.2. Optimization of DPV Parameters

Basic parameters of DPV, as scan rate, pulse height, and pulse width, were optimized directly for DAN measurement. These parameters were studied in the following ranges: 10–100 mV s^−1^ (*v*), 10–100 mV (pulse height), and 20–100 ms (pulse width). The values chosen for all following measurements are summarized in Table 1. As expected, the optimum DPV parameters are the same for HMDE and both types of AgSAE. The values chosen for BiFE utilization differ partly only.

**Figure 6:**
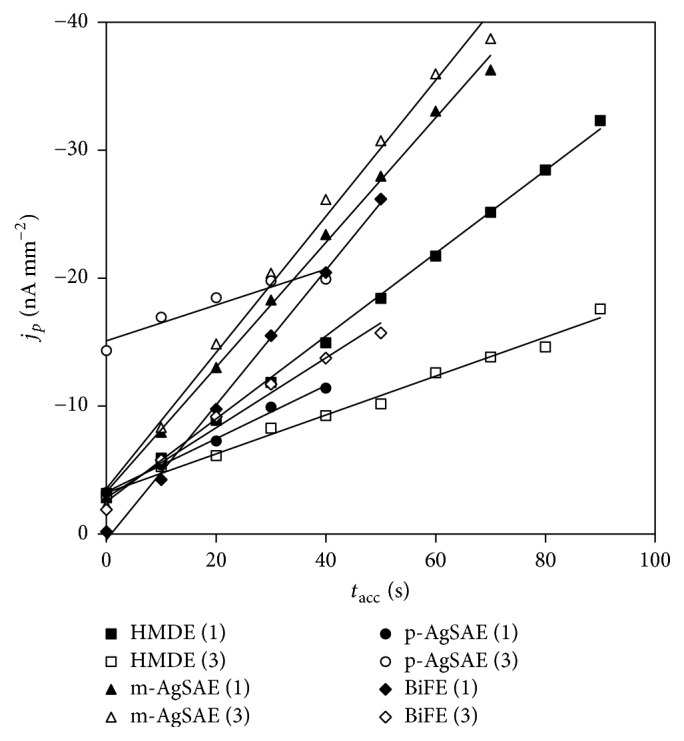
Dependence of *j*_*p*_ of DAN signals on *t*_acc_ obtained on all tested electrodes. Method: DPAdSV; electrolyte: BRB (pH 6.0 and 5.0 (BiFE)); *E*_in_ = +200 mV; *E*_fin_ = −1100 mV; *v* = 40 mV s^−1^;* pulse height* = −60 mV and −50 mV (BiFE);* pulse width* = 40 ms and 60 ms (BiFE); *E*_acc_ = +200 mV; *t*_acc_ = 0–90 s (HMDE), 0–70 s (m-AgSAE), 0–40 s (p-AgSAE), and 0–50 s (BiFE); *c*_DAN_ = 2.0 × 10^−7^ mol L^−1^; (1) peak 1; (3) peak 3.

Next parameters as potential (*E*_acc_) and time of accumulation (*t*_acc_) were tested by the analysis of 2.0 × 10^−7^ mol L^−1^ DAN solution. The value of +200 mV was confirmed as the optimal *E*_acc_ for all used electrodes. In Figure 6 the obtained dependence of *I*_*p*_ on *t*_acc_ is shown. It is evident that *j*_*p*_ of DAN signals 1 and 3 are strongly dependent on *t*_acc_ and they are directly proportional to the applied *t*_acc_. It corresponds with the above-mentioned results proving influence of adsorption on all registered electrode processes.

The achieved parameters (slopes, intercepts, and *r*) of the dependence of *I*_*p*_ on *t*_acc_ are summarized in Table 5. The values of slopes (sensitivities) show that the accumulation much more influences the first DAN peaks. It is in accordance with above described effect of adsorption of DAN on the electrode surfaces (except m-AgSAE, wherein very strong influence of adsorption was confirmed for both observed signals). The highest increase of *j*_*p*_ with *t*_acc_ was found for m-AgSAE and surprisingly also for BiFE, which provided diffusion-controlled electrode processes with relatively weak adsorption influence. Both signals recorded with p-AgSAE provided only low increases of *j*_*p*_ with increasing *t*_acc_.

#### 3.3.3. Determination of DAN in Model Solution

**Figure 7:**
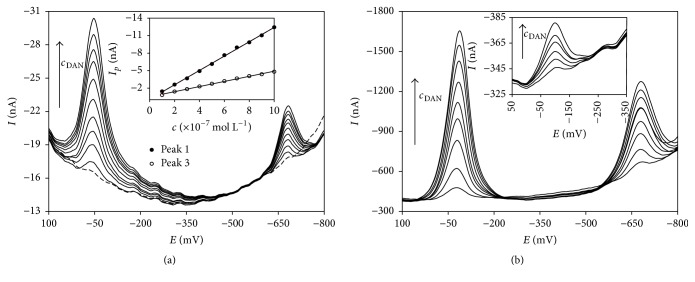
DP voltammograms of DAN in dependence on *c*_DAN_ obtained on m-AgSAE (a) and BiFE (b). DPAdSV (a) and DPV (b); electrolyte: BRB (pH 6.0 (a) and 5.0 (b)); *E*_in_ = +200 mV; *E*_fin_ = −1100 mV; *v* = 40 mV s^−1^;* pulse height* = −60 mV (a) and −50 mV (b);* pulse width* = 40 ms (a) and 60 ms (b); *E*_acc_ = +200 mV; *t*_acc_ = 20 s (a) and 0.0 s (b); *c*_DAN_ = 1.0 × 10^−7^–1.0 × 10^−6^ mol L^−1^ (a) and 1.0 × 10^−6^–9.0 × 10^−6^ mol L^−1^ (b).* Inset (a)*: dependence of *I*_*p*_ on *c*_DAN_ (m-AgSAE) for both DAN signals.* Inset (b)*: DP voltammograms of DAN in dependence on *c*_DAN_ (BiFE, DPAdSV, *t*_acc_ = 40 s, and *c*_DAN_ 1.0 × 10^−8^–6.0 × 10^−8^ mol L^−1^).

DPV as well as DPAdSV was applied for measurement of various types of DAN concentration dependence in model solutions.  Figure 7 presents voltammograms obtained with m-AgSAE (a) and BiFE (b). Both observed DAN responses increase linearly with DAN content. It is demonstrated also by the inset of Figure 7(a) recorded using m-AgSAE (DPAdSV, *t*_acc_ = 20 s) in concentration range from 1.0 × 10^−7^–1.0 × 10^−6^ mol L^−1^.  Figure 7(b) shows successful application of DPV for higher DAN concentration levels (1.0 × 10^−6^–9.0 × 10^−6^ mol L^−1^) as well as of DPAdSV for lower ones (1.0 × 10^−8^–6.0 × 10^−8^ mol L^−1^, *t*_acc_ = 40 s) on the example of BiFE.  Table 6 summarizes the parameters of the linear dependence of *j*_*p*_ on DAN concentration (for peak 1) in the range from 1.0 × 10^−6^ to 1.0 × 10^−7^ mol L^−1^ obtained by DPV (i.e., *t*_acc_ = 0.0) using all investigated electrodes. It was proved that first DAN signal is more suitable for its quantitative determination because it is more intensive and better evaluable applying all tested electrodes. The highest value of slope (−31.43 ± 0.44) nA L mm^−2^ mol^−1^ was achieved with m-AgSAE which indicates the highest sensitivity of this electrode. Conversely, BiFE seems to be the least sensitive from this comparison (−22.13 ± 0.65) nA L mm^−2^ mol^−1^.

The situation was significantly changed by insertion of an accumulation step. As it was stated above (Table 5), the accumulation influences the most positively reduction signal 1 registered on BiFE. Therefore, very low values of LOD (compared with HMDE [49]) were obtained for BiFE (5.0 × 10^−10^ mol L^−1^) as well as for m-AgSAE (7.5 × 10^−10^ mol L^−1^) using DPAdSV. Both electrodes provided also very wide linear dynamic range (LDR). However, it should be recalled that the electrode surface of BiFE is much larger than in case of m-AgSAE. Statistical parameters for all developed methods of DAN determination using particular tested electrodes are summarized in Table 7. The highest LOD was achieved for p-AgSAE (2.0 × 10^−8^ mol L^−1^, *t*_acc_ = 100 s) as well as a bit narrower LDR.

#### 3.3.4. Determination of DAN in Model Drinking Water Sample

**Figure 8:**
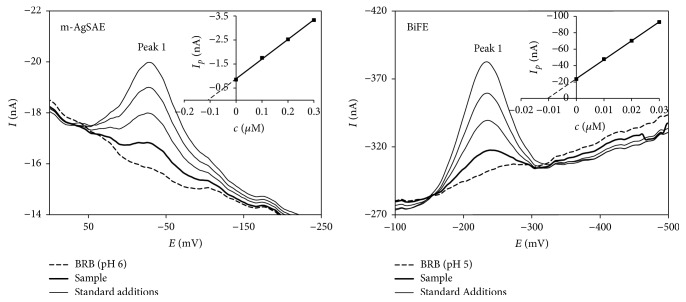
DP voltammograms of DAN determination in model samples using standard addition method obtained on m-AgSAE and BiFE. Method: DPAdSV; electrolyte: BRB (pH 6.0 (m-AgSAE) and 5.0 (BiFE);, *E*_in_ = +200 mV; *E*_fin_ = −1100 mV; *v* = 40 mV s^−1^;* pulse height* = −60 mV (m-AgSAE) and −50 mV (BiFE);* pulse width* = 40 ms (m-AgSAE) and 60 ms (BiFE); *E*_acc_ = +200 mV; *t*_acc_ = 20 s (m-AgSAE) and 40 s (BiFE); *c*_DAN_ = 1.0 × 10^−7^ mol L^−1^ (m-AgSAE) and 1.0 × 10^−8^ mol L^−1^ (BiFE).* Insets*: graphical evaluation of analyses.

As the last, the applicability of developed methods for DAN determinations was tested using all investigated electrodes via analysis of drinking water spiked with two different contents of DAN. Standard addition method with at least 2-3 standard additions was applied in case of each electrode. Examples of the measurements realized with m-AgSAE and BiFE are shown in Figure 8 always with the graphical evaluation of standard addition method. Every analysis was five times repeated and the obtained results as averages with the appropriated confidence intervals and relative standard deviations (RSD_5_) are presented in Table 8. These results confirmed that the determination of DAN with all tested electrodes can give true and well repeatable (RSD_5_ < 3.0%) results. In the cases of p-AgSAE higher concentration levels of DAN in model samples were analyzed with respect to its higher LODs in comparison with m-AgSAE as well as BiFE.

## 4. Conclusion

This work was focused not only on the development of voltammetric methods for DAN determination, but also on the comparison of electrochemical properties of AgSAEs and BiFE as the most commonly used replacements for mercury electrodes.

It was found that BiFE provides somewhat narrower potential window in the cathodic area than HMDE and also than both m-AgSAE and p-AgSAE. Moreover, handling with this electrode is more complicated and time-consuming due to the necessity of repeated Bi film preparation. Nevertheless, the voltammetric behavior of DAN was very similar using all electrodes and it provided the same reduction signals as on HMDE. Some differences in controlling processes were found. While the adsorption-controlled processes were confirmed for electrodes with liquid mercury surface (i.e., HMDE and m-AgSAE), diffusion was proved as controlling mechanism using p-AgSAE and BiFE.

Voltammetric methods for DAN determination using DPV in connection with all tested electrodes were developed. It was proved that both modifications of AgSAEs as well as BiFE can be used for DAN determination and that the application of AdSV improves the reached LODs. Finally, all tested electrodes were successfully applied for DAN determination in model solutions of spiked drinking water.

## Figures and Tables

**Table 1:** Optimized DPV parameters for DAN determination.

Parameter	HMDE	m-AgSAE	p-AgSAE	BiFE
pH	6.0	6.0	6.0	5.0
*v* [mV s^−1^]	40	40	40	40
Pulse height [mV]	−60	−60	−60	−50
Pulse width^*∗*^ [ms]	40	40	40	60
*E*_acc_ [mV]	+200	+200	+200	+200

^*∗*^Current values were recorded and averaged in the last 20 ms of the pulse duration.

**Table 2:** Peak positions of DAN in BRB of pH 6.0 recorded on all tested electrodes using CV (*E*_in_ = +200 mV, *E*_fin_ = −1300 mV, *v* = 100 mV s^−1^, and *c*_DAN_ = 5.0 × 10^−6^ mol L^−1^).

Electrode	*E*_*p*1_ [mV]	*E*_*p*2_ [mV]	*E*_*p*3_ [mV]
HMDE	−220	−520	−895
m-AgSAE	−210	−490	−850
p-AgSAE	−95	−380	−717
BiFE	−276	—	−873

**Table 3:** Parameters of the dependences of *E*_*p*_ on pH of the electrolyte (CV, BRB (pH 2.0–12), *E*_in_ = +200 mV, *E*_fin_ = −1300 mV, *v* = 100 mV s^−1^, and *c*_DAN_ = 5.0 × 10^−6^ mol L^−1^).

Electrode	Peak	Slope [mV]	Intercept [mV]	*r*
HMDE	1	(−51.16 ± 0.62)	(74.6 ± 4.8)	0.99931
	3	(−59.8 ± 3.8)	(−525 ± 25)	0.986
m-AgSAE	1	(−51.8 ± 1.9)	(83 ± 14)	0.9941
	3	(−75.4 ± 3.5)	(−408 ± 23)	0.9927
p-AgSAE	1	(−43.8 ± 1.7)	(197 ± 13)	0.9936
	3	(−51.2 ± 2.7)	(−347 ± 17)	0.9909
BiFE	1	(−41.1 ± 3.8)	—	0.972
	3	(−95 ± 10)	(−318 ± 50)	0.977

**Table 4:** Parameters of the dependences of *I*_*p*_ on *v* (CV, BRB (pH 5.0 (BiFE), 6.0 (the other electrodes)), *E*_in_ = +200 mV, *E*_fin_ = −1300 mV, *v* = 50–500 mV s^−1^, and *c*_DAN_ = 5.0 × 10^−6^ mol L^−1^). The confidence intervals are expressed on level of significance *α* = 0.05.

Electrode	Peak	Dependence *I*_*p*__*v* *I*_*p*__*v*^1/2^ log(*I*_*p*_)_log(*v*)	Slope [nA s mV^−1^] [nA s^1/2^ mV^−1/2^] log([nA s mV^−1^])	Intercept [nA] [nA] log([nA])	*r*
HMDE	1	*I*_*p*__*v*	(−0.1502 ± 0.0025)	(−2.93 ± 0.78)	0.9988
		log(*I*_*p*_)_log(*v*)	(0.868 ± 0.023)	(−0.466 ± 0.055)	0.9971
	3	*I*_*p*__*v*	(−1.6040 ± 0.0026)	(−12.42 ± 0.82)	0.9989
		log(*I*_*p*_)_log(*v*)	(0.687 ± 0.018)	(0.090 ± 0.044)	0.9970

m-AgSAE	1	*I*_*p*__*v*	(−0.2323 ± 0.0069)	(−9.7 ± 2.1)	0.9965
		log(*I*_*p*_)_log(*v*)	(0.835 ± 0.011)	(−0.160 ± 0.026)	0.99930
	3	*I*_*p*__*v*	(−0.1149 ± 0.0052)	(−6.7 ± 1.6)	0.9948
		log(*I*_*p*_)_log(*v*)	(1.248 ± 0.022)	(−1.546 ± 0.052)	0.9988

p-AgSAE	1	*I*_*p*__*v*^1/2^	(−5.73 ± 0.16)	(11.3 ± 2.7)	0.9969
		log(*I*_*p*_)_log(*v*)	(0.624 ± 0.022)	(−0.398 ± 0.051)	0.9952
	3	*I*_*p*__*v*^1/2^	(−5.46 ± 0.32)	(10.3 ± 5.4)	0.9863
		log(*I*_*p*_)_log(*v*)	(0.625 ± 0.035)	(−0.377 ± 0.082)	0.988

BiFE	1	*I*_*p*__*v*^1/2^	(−246.6 ± 4.8)	(385 ± 79)	0.9985
		log(*I*_*p*_)_log(*v*)	(0.5632 ± 0.0081)	(2.192 ± 0.019)	0.99921
	3	*I*_*p*__*v*^1/2^	(−40.4 ± 2.9)	—	0.987
		log(*I*_*p*_)_log(*v*)	(0.506 ± 0.067)	(1.577 ± 0.089)	0.979

**Table 5:** Parameters of the dependences of *j*_*p*_ on *t*_acc_ (DPAdSV, BRB (pH 5.0 (BiFE), 6.0 (the other electrodes)), *E*_in_ = +200 mV, *E*_fin_ = −1300 mV, *v* = 40 mV s^−1^, *pulse height* = −50 mV (BiFE) and −60 mV (the other electrodes), *pulse width* = 60 ms (BiFE) and 40 ms (the other electrodes), *E*_acc_ = +200 mV, *t*_acc_ = 0–90 s (HMDE), 0–70 s (m-AgSAE), 0–40 s (p-AgSAE), and 0–50 s (BiFE), and *c*_DAN_ = 2.0 × 10^−7^ mol L^−1^); *α* = 0.05.

Electrode	Peak	Slope [nA mm^−2^ s^−1^]	Intercept [nA mm^−2^]	*r*
HMDE	1	(−0.3238 ± 0.0047)	(−2.51 ± 0.25)	0.99910
	3	(−0.1519 ± 0.0058)	(−3.23 ± 0.31)	0.9942
m-AgSAE	1	(−0.4872 ± 0.0099)	(−3.30 ± 0.41)	0.9987
	3	(−0.532 ± 0.020)	(−3.55 ± 0.83)	0.9959
p-AgSAE	1	(−0.2083 ± 0.0088)	(−3.29 ± 0.21)	0.9973
	3	(−0.140 ± 0.025)	(−15.09 ± 0.62)	0.954
BiFE	1	(−0.527 ± 0.012)	(0.44 ± 0.36)	0.9989
	3	(−0.273 ± 0.020)	(−2.86 ± 0.60)	0.9900

**Table 6:** Parameters of the dependences of *j*_*p*_ on *c*_DAN_ (DPV, BRB (pH 5.0 (BiFE), 6.0 (the other ones)), *E*_in_ = +200 mV, *E*_fin_ = −1300 mV, *v* = 40 mV s^−1^, *pulse height* = −50 mV (BiFE), −60 mV (the other ones), *pulse width* = 60 ms (BiFE), 40 ms (the other ones), and *c*_DAN_ = 1.0 × 10^−6^–1.0 × 10^−7 ^mol L^−1^); *α* = 0.05.

Electrode	Slope [nA L mm^−2^ mol^−1^]	Intercept [nA mm^−2^]	*r*
HMDE	(−28.87 ± 0.44)	(−2.7 ± 1.8)	0.99951
m-AgSAE	(−31.43 ± 0.44)	(−3.6 ± 2.6)	0.99929
p-AgSAE	(−25.6 ± 1.3)	(−7.5 ± 5.0)	0.9952
BiFE	(−22.13 ± 0.65)	(5.0 ± 3.5)	0.9971

**Table 7:** Statistical parameters of DAN determination using all tested working electrodes (DPAdSV, BRB (pH 5.0 (BiFE), 6.0 (the other ones)), *E*_in_ = +200 mV, *E*_fin_ = −1300 mV, *v* = 40 mV s^−1^, *pulse height* = −50 mV (BiFE), −60 mV (the other ones), *pulse width* = 60 ms (BiFE), 40 ms (the other ones), *E*_acc_ = +200 mV, and *t*_acc_ = 0–100 s (in dependence on *c*_DAN_)).

Electrode	Area [mm^2^]	Lower limit [mol L^−1^]	Upper limit [mol L^−1^]	LOD [mol L^−1^]	LOQ [mol L^−1^]	RSD_11_^*∗*^ [%]
HMDE (see [49])	—	—	—	2.1 × 10^−10^	7.0 × 10^−10^	—
m-AgSAE	0.39	3.0 × 10^−9^	3.0 × 10^−5^	7.5 × 10^−10^	2.5 × 10^−9^	2.2
p-AgSAE	0.28	1.0 × 10^−7^	5.0 × 10^−5^	2.0 × 10^−8^	7.0 × 10^−8^	2.9
BiFE	7.07	1.0 × 10^−9^	5.0 × 10^−5^	5.0 × 10^−10^	1.9 × 10^−9^	2.3

^*∗*^ *c*_DAN_ = 5 × 10^−7^ mol L^−1^.

**Table 8:** Repeatability and recovery of DAN determination in spiked drinking water using p-AgSAE, m-AgSAE, and BiFE (DPAdSV, BRB (pH 5.0 (BiFE), 6.0 (AgSAEs)), *E*_in_ = +200 mV, *E*_fin_ = −1300 mV, *v* = 40 mV s^−1^, *pulse height* = −50 mV (BiFE), −60 mV (AgSAEs), *pulse width* = 60 ms (BiFE), 40 ms (AgSAEs), *E*_acc_ = +200 mV, and *t*_acc_ = 0–50 s (in dependence on *c*_DAN_)); *α* = 0.05.

Electrode	Added [mol L^−1^]	Found [mol L^−1^]	Recovery [%]	RSD_5_ [%]
m-AgSAE	1.0 × 10^−7^	(1.010 ± 0.016) × 10^−7^	99.7–104.0	2.3
	1.0 × 10^−8^	(1.010 ± 0.022) × 10^−8^	99.7–103.2	1.9
p-AgSAE	2.0 × 10^−6^	(2.020 ± 0.024) × 10^−6^	99.6–104.4	1.8
	2.0 × 10^−7^	(1.021 ± 0.022) × 10^−7^	99.8–104.9	2.5
BiFE	1.0 × 10^−7^	(1.010 ± 0.014) × 10^−7^	99.6–104.0	2.1
	1.0 × 10^−8^	(1.021 ± 0.029) × 10^−8^	99.7–104.8	2.9
